# Discovery of prenylated flavonoids with dual activity against influenza virus and Streptococcus pneumoniae

**DOI:** 10.1038/srep27156

**Published:** 2016-06-03

**Authors:** Ulrike Grienke, Martina Richter, Elisabeth Walther, Anja Hoffmann, Johannes Kirchmair, Vadim Makarov, Sandor Nietzsche, Michaela Schmidtke, Judith M. Rollinger

**Affiliations:** 1 Department of Pharmacognosy, Faculty of Life Sciences, University of Vienna, Althanstraße 14, 1090 Vienna, Austria; 2 Jena University Hospital, Department of Virology and Antiviral Therapy, Hans-Knoell-Strasse 2, 07745 Jena, Germany; 3 University of Hamburg, Center for Bioinformatics, Bundesstrasse 43, 20146 Hamburg, Germany; 4 A.N. Bakh Institute of Biochemistry RAS, Leninsky prospekt, 33, build. 2, Moscow, 119071, Russia; 5 Jena University Hospital, Center of Electron Microscopy, Ziegelmuehlenweg 1, 07743 Jena, Germany

## Abstract

Influenza virus neuraminidase (NA) is the primary target for influenza therapeutics. Severe complications are often related to secondary pneumonia caused by *Streptococcus pneumoniae* (pneumococci), which also express NAs. Recently, a NA-mediated lethal synergism between influenza A viruses and pneumococci was described. Therefore, dual inhibitors of both viral and bacterial NAs are expected to be advantageous for the treatment of influenza. We investigated the traditional Chinese herbal drug sāng bái pí (mulberry root bark) as source for anti-infectives. Two prenylated flavonoid derivatives, sanggenon G (**4**) and sanggenol A (**5**) inhibited influenza A viral and pneumococcal NAs and, in contrast to the approved NA inhibitor oseltamivir, also planktonic growth and biofilm formation of pneumococci. Evaluation of **27** congeners of **5** revealed a correlation between the degree of prenylation and bioactivity. Abyssinone-V 4′-methyl ether (**27**) inhibited pneumococcal NA with IC_50_ = 2.18 μM, pneumococcal growth with MIC = 5.63 μM, and biofilm formation with MBIC = 4.21 μM, without harming lung epithelial cells. Compounds **5** and **27** also disrupt the synergism between influenza A virus and pneumococcal NA *in vitro*, hence functioning as dual-acting anti-infectives. The results warrant further studies on whether the observed disruption of this synergism is transferable to *in vivo* systems.

Influenza, an acute viral infection of the respiratory tract, causes about 3 to 5 million cases of severe illness, and about 250,000 to 500,000 deaths every year worldwide^1^. Influenza A virus (IAV) infections are often associated with secondary complications caused by bacterial pathogens, most commonly *Streptococcus pneumoniae (S. pneumoniae)*^2^. There is a complex interplay of IAV and *S. pneumoniae*, which have developed a lethal synergistic strategy mediated by neuraminidases (NAs)^2^,^3^,^4^. NAs are present on both pathogens. The viral NA facilitates viral reproduction and spread^5^,^6^ and is an established drug target. All pneumococci produce NA NanA to access receptors on lung epithelial cells^2^,^7^,^8^. By cleaving sialic acids from cell surface glycoproteins, viral NA provides attachment receptors and nutrients for *S. pneumoniae* colonization and growth^4^,^9^. In turn, NanA has recently been reported to contribute to the synergism by supporting viral release when added upon infection^3^. Since bacterial superinfection is a major factor in influenza mortality and viral and bacterial NAs are structurally related^10^,^11^,^12^, dual inhibition of both NAs presents an innovative strategy for therapy^8^,^13^. Moreover, *S. pneumoniae* forms a bacterial biofilm which is composed of an accumulation of bacteria covered by an extracellular matrix promoting a chronic disease progress^14^. Hence, prevention and control of these infections is a challenge for the development of anti-infective agents.

In the last few years, several studies have reported the discovery of influenza virus neuraminidase inhibitors (NAIs) isolated from natural sources^13^,^15^,^16^, whereby flavonoids are the most thoroughly investigated class of compounds^16^,^17^. Various studies have focused on their ability to inhibit either bacterial or viral NAs^16^. However, some of them reported a considerable quenching effect or self-fluorescence (self-FL) of flavonoids, causing false-positive results in the commonly used enzyme-based NA inhibition assays^18^,^19^. Thus, bioactivities reported for flavonoids with these testing systems should be considered with caution.

During an ongoing screening campaign for natural products active on IAV and pneumococcal NAs^15^,^20^,^21^, we identified the di-prenylated flavone artocarpin as dual NAI, with a distinct inhibitory activity on pneumococcal growth and biofilm formation^8^,^13^. Prenylated flavonoids occur in a comparably small number of plant families, such as Fabaceae and Moraceae.

In this follow-up study, the root bark of the white mulberry tree (*Morus alba* L.; Moraceae) was selected as a plant source containing constituents (primarily flavonoids) that share characteristic prenyl features with the previously identified antipneumococcal NAI artocarpin^15^,^21^. In traditional Chinese medicine (TCM), white mulberry root bark is known under the name sāng bái pí, which “drains the lungs, especially heat in the lungs, thereby alleviating cough and wheezing”, suggesting a beneficial effect on symptoms related to influenza and pneumonia^22^. In addition to phytochemical and biological investigations of anti-infective effects of sāng bái pí constituents, this work describes the evaluation of a series of sanggenol A (**5**) congeners for their ability to inhibit viral and bacterial NAs as well as their antiviral and antibacterial potency. Thereby, several complementary assays were used in combination to confirm bioactivity. The study also analyses whether the investigated compounds prevent synergism of IAV and pneumococcal NanA *in vitro*. Finally, results from in depth electron-microscopic investigations of abyssinone-V 4′-methyl ether (**27**), one of the most potent bacterial NAIs, which also inhibits planktonic growth of pneumococci and their biofilm formation, are reported.

## Results

Starting from a methanol extract (MAE) and a fraction enriched with prenylated constituents (MAF), seven mulberry root bark constituents were isolated by separation techniques including liquid-liquid partition, column chromatography, and high-speed counter current chromatography (HSCCC).

**Figure 1:**
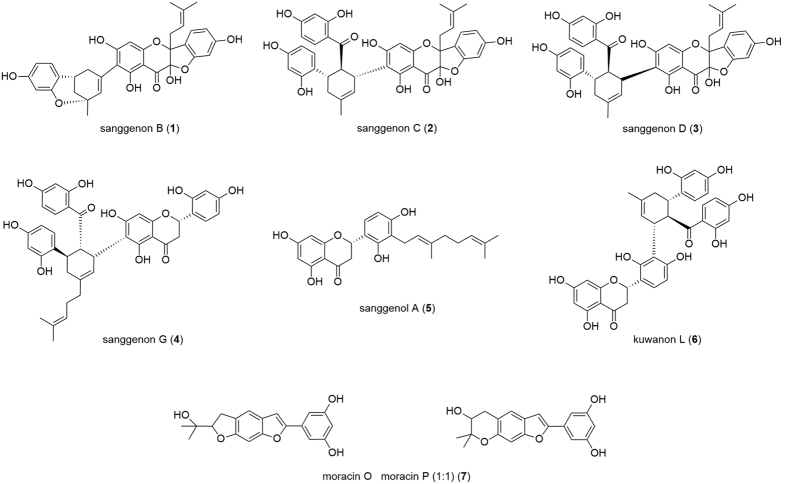
Chemical structures of isolated mulberry root bark constituents.

By using LC-MS analysis and NMR experiments, and by comparison with earlier studies^23^,^24^, the constituents were identified as sanggenon B (**1**), sanggenon C (**2**), sanggenon D (**3**), sanggenon G (**4**), sanggenol A (**5**), kuwanon L (**6**), and the 1:1 mixture of moracin O and moracin P (**7**) (Fig. 1). The sanggenons (**1**–**4**) and **6** can be classified as Diels-Alder adducts based on a mono- or di-prenylated flavonoid unit, whereby one prenyl residue is linked to a chalcon unit via a cyclohexane ring. Compound **5** is a di-prenylated (geranyl) tetrahydroxyflavane, and **7** is a 1:1 mixture of tricyclic fused ring systems having a benzofuran scaffold.

For providing a better basis for statements concerning the traditional use of sāng bái pí, an aqueous decoction (MAD) was prepared. By HPLC comparison, the presence of compounds **1** to **7** was confirmed in all three multicomponent mixtures (MAE, MAF, MAD; Supporting Fig. S1).

### Antimicrobial investigations of the *M. alba* root bark extract, enriched fraction and pure constituents

The inhibitory potential of *M. alba* samples against *S. pneumoniae* and influenza virus NAs was evaluated in fluorescence (FL)-based enzyme inhibition assays and (under physiologically more relevant conditions) in hemagglutination (HA)-based assays with human erythrocytes (referred to as FL and HA assay, respectively; Table 1). The readout of the FL assay is based on the quantification of the FL signal released after cleavage of the sialic acid-containing synthetic substrate (MUNANA) by NAs of the H1N1 influenza virus strains A/WSN/1933 (WSN/33^25^) and A/Jena/8178/2009 (8178/09; A(H1N1)pdm09 strain), and the recombinant NanA of pneumococcal strain DSM20566 (rNanA) at pH 6.0^13^. In order to rule out false positive or negative results, all samples were checked for FL quenching and self-FL as described previously (Supplementary Table S1)^13^. Since neither WSN/33 nor 8178/09 NA did elute agglutinated human erythrocytes, the HA assay was performed with influenza virus strain A/Jena/5258/2009 (5258/09; A(H1N1)pdm09 strain) in parallel to the NA-containing precipitate of strain DSM20566. Before evaluation of bioactivity, potential assay interferences, e.g. erythrocyte lysis, hemagglutination of erythrocytes or, in the case of viral NAs, prevention of virus-induced hemagglutination by test compounds were ruled out (Supplementary Table S2).

As expected, oseltamivir, used as a dual-acting^8^,^20^ control, inhibits IAV NAs as well as pneumococcal NAs (with a much lower activity) in the FL and HA assay. No assay interference was observed.

At the multi-component level (MAE and MAF), inhibition of viral NA was weak (Table 1 and Supplementary Table S3) or could not be investigated due to problems with assay readout (Supplementary Tables S1 and S2), which were most likely caused by the presence of assay-interfering constituents in the complex mixtures. MAE inhibited bacterial NA in the FL assay with IC_50_ = 36.6 μg/mL, which however could not be confirmed by the HA assay. Interestingly, the MAE fraction MAF, enriched with prenylated constituents, showed distinct inhibitory activity against pneumococcal NA in the FL assay (IC_50_ 20.5 μg/mL) and the HA assay (MIC 10.0 μg/mL) (Table 1).

Most of the compounds isolated from MAF exerted stronger inhibitory activities against pneumococcal NA (lower values indicate stronger activity) as compared to viral NA. However, interpretation of the readouts obtained with viral NA was often hindered by self-FL in the FL assay (Supplementary Table S1) or by erythrocyte lysis and prevention of virus-induced hemagglutination in the HA assay (Supplementary Table S2). The most pronounced inhibition of the pneumococcal NA in the FL assay was observed for **3**, **4**, **5**, and **6**, with IC_50_ values of 3.1, 2.9, 6.2, and 8.3 μM, respectively. For these compounds the inhibitory effects could be confirmed in the HA assay, with MICs of 5.4 (**4**) and 31.6 μM (all others).

Most compounds were well tolerated by Madin-Darby canine kidney (MDCK) and A549 lung epithelial cells, whereby results determined in lung epithelial cells might be physiologically more relevant (Table 2).

The antiviral potential of the multi-component mixtures and pure constituents from *M. alba* was demonstrated in cytopathic effect (CPE) inhibitory assays with the IAV strains WSN/33 and/or 8178/09 in MDCK cells. MAE inhibited the CPE of 8178/09 or WSN/33 by 50% at concentrations of 29.7 μg/mL and 9.33 μg/mL (Table 2) whereas MAF acted at 76.6 μg/mL and 23.0 μg/mL (Supplementary Table S3), respectively. Some of its isolates reduced the 8178/09-induced (**2** and **4**) and/or WSN/33-induced CPE (**4**, **5**, and **6**) in MDCK cells, whereas the other constituents (i.e. **1**, **3**, and **7**) showed no activity in this phenotypic assay.

Concerning the general antibacterial activity of *M. alba* constituents on *S. pneumoniae*, with the exception of **1**, **6**, and **7**, all isolated compounds inhibited the planktonic growth and biofilm formation of DSM20566 at concentrations lower than 10 μM by 90% (Table 2).

All these results together show that **4** and **5** have the most promising antiviral and antibacterial profile, which correlates with their inhibitory activity against both viral and pneumococcal NAs (with the latter being more susceptible). Since **4** exerted stronger assay interference phenomena than the geranylated flavanone **5**, the latter was selected for further investigations.

### Evaluation of anti-infective properties of congeners of sanggenol A (compound 5)

**Figure 2:**
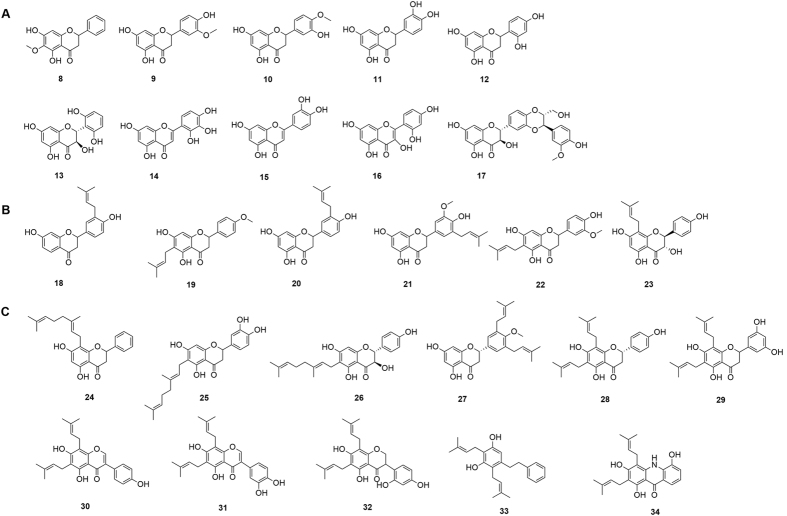
Chemical structures of non- (**A**), mono- (**B**), and di-prenylated (**C**) congeners of **5**.

In search for further compounds with inhibitory activity on IAV and *S. pneumoniae*, a selection of off-the-shelf congeners of **5** (i.e. non-prenylated, mono-prenylated and di-prenylated compounds) (Fig. 2) was tested for their inhibitory activity on viral and bacterial NA.

In general, the tested congeners of **5** showed higher activity on the bacterial than the viral enzyme (Table 1). The structure-activity landscape is comparably flat, with the majority of tested compounds active on bacterial NA in the low micromolar range. Among the tested congeners only a few compounds can be considered as inactive, i.e. **15**, **22** and **23** (FL assay). Activity appears to increase with the degree of prenylation and hence, hydrophobicity. In particular the di-prenylated compounds **24** and **27** exerted strong bacterial NA inhibitory activities in the FL as well as the HA assay.

Alignment of bioactive congeners on their flavonoid scaffold suggests that bulky substituents may be attached to several positions of the scaffold without a consistent loss of bioactivity (data not shown). This renders a common binding mode for this chemical series in proximity of the substrate binding site improbable. No co-crystal of a flavonoid bound with NA has been reported to date, to the best of our knowledge. From a docking experiment with compound **5** on a homology model of NanA (using Glide^26^), varying orientations of the ligand were obtained, some of which with the flavonoid scaffold placed at the substrate binding site and others with the lipophilic geranyl moiety oriented toward it (data not shown). The results from this docking experiment were inconclusive, and all observations considered together indicate that **5** and its congeners are unlikely to share a single binding mode. Rather, the observed inhibitory activity may be the result of interactions related to the compatibility of the physicochemical properties of ligand and protein, with hydrophobic interactions playing a significant role.

With the exception of **18** (WSN/33 and 8178/09) and **23** (8178/09), none of the tested compounds inhibited the virus-induced CPE in MDCK cells at non-cytotoxic concentrations. However, compound **27** significantly reduced the virus yield of untreated virus control, set as 100%, to 7.4 ± 1.2% in A549 cells after treatment with 50 μM of **27**. This concentration could not be tested in MDCK cells due to cytotoxicity concerns. In accordance with mentioned antibacterial NA activities, strong anti-pneumococcal effects were observed especially for di-prenylated congeners of compound **5**.

### Confirmation of biofilm inhibition by compound **27** using scanning electron microscopy studies

**Figure 3:**
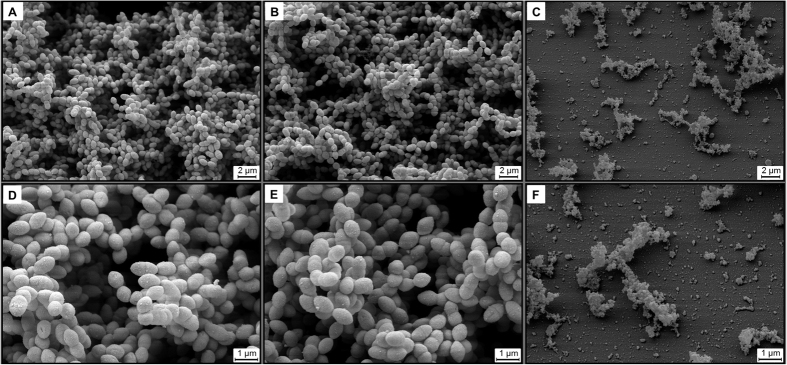
Effect of oseltamivir and compound 27 on pneumococcal biofilm formation. Three hours after inoculation of bacteria onto glass slides in 24-well plates the supernatant was replaced with fresh medium containing no inhibitor (**A,D**), 10 μM of oseltamivir (**B,E**) or 10 μM of compound **27** (**C,F**). Bacteria were incubated at 37 °C and 5% CO_2_ for 7 days and afterwards analysed by scanning electron microscopy. The experiment was performed twice. Representative photographs are shown.

To visualise the effect of di-prenylated congeners of **5** on biofilm formation of pneumococci, scanning electron microscopy was applied. After establishing the experimental conditions for biofilm production of the pneumococcal strain DSM20566 on glass slides, bacteria were treated with **27**, one of the strongest inhibitors of bacterial NA and bacterial biofilm formation. DSM20566 pneumococci were treated with medium (mock-treated) and 10 μM of oseltamivir (virus-specific NAI) or **27**, for 7 days, starting 3 h after inoculation of bacteria (the time necessary for the attachment of pneumococci to glass slides). At the end of the incubation time a distinct pneumococcal biofilm was formed on mock-treated slides with typical bacteria clusters crisscrossed by water channels (Fig. 3A,D). Oseltamivir treatment did not impair biofilm formation (Fig. 3B,E). In contrast, treatment of pneumococci with compound **27** completely prevented biofilm formation and induced the death of bacteria (Fig. 3C,F).

**Figure 4:**
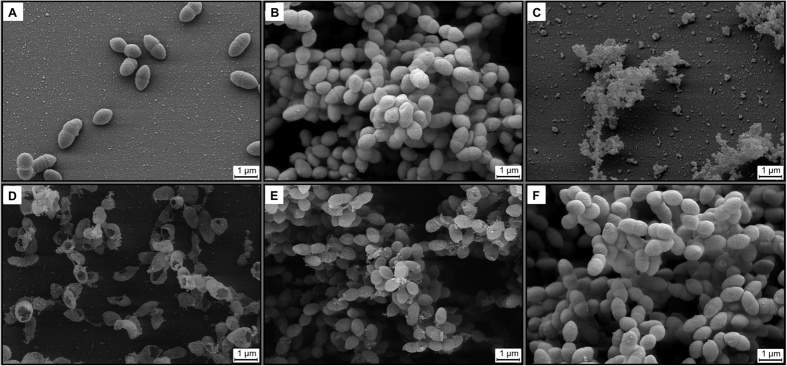
Time-dependent inhibition of pneumococcal biofilm formation by compound **27**. Three hours after inoculation of bacteria in 24-well plates, bacteria were fixed (control, (**A**)). The supernatant of other wells was replaced by fresh medium containing no inhibitor (**B**) or 10 μM of **27** (**C–F**). Three (**C**), six (**D**), nine (**E**) or 48 h (**F**) after inoculation, **27** was added to the medium to reach a concentration of 10 μM in the supernatant. Bacteria were incubated at 37 °C and 5% CO_2_ for 7 days and afterwards analysed by scanning electron microscopy.

The time-dependent antibacterial effect of **27** was studied to determine whether the compound acts on bacteria during biofilm formation and/or the developed pneumococcal biofilm. Therefore, treatment with 10 μM of **27** was started (i) directly after the attachment of single pneumococci to the glass surface at three hours after inoculation of bacteria (an example for attached bacteria is shown for control in Fig. 4A), (ii) at two time points during biofilm growth (six or nine hours after inoculation of bacteria), or (iii) when the biofilm was formed (48 hours after inoculation of the bacteria). After 7 days of incubation a biofilm was formed in mock-treated wells, confirming the results of the first experiment (Fig. 4B). A strong effect was confirmed when **27** was added three hours after inoculation of bacteria (Fig. 4C). When the treatment with **27** started six hours after inoculation of bacteria, no biofilm was formed (Fig. 4D). The addition of compound **27** nine hours after bacteria inoculation affected biofilm formation weakly (Fig. 4E). Late treatment (i.e. 48 h after bacteria inoculation) had no effect (Fig. 4F). The scanning electron microscopy studies confirmed the inhibitory effect of **27** on biofilm formation of *S. pneumoniae*.

### Compounds 5 and 27 disrupt the synergism between viral and *S. pneumoniae* NA *in vitro*

Due to structural and functional similarities to viral NA^12^,^16^, the ubiquitously expressed NanA in *S. pneumoniae*^7^,^8^,^27^ supports influenza virus release from infected cells^3^. Moreover, results from previous studies showed that bacterial NA rescues influenza virus replication from inhibition by zanamivir^3^,^28^. In contrast, both **5** and **27** (50 µM) reduce the viral yield in A549 cells in the presence of NanA of DSM20566 from 100 to 31.8 ± 4.5% and 7.4 ± 1.2%. These results are in good agreement with the virus yield reduction in the absence of NanA (reduction to 5.1 ± 1.8% and 3.2 ± 1.4%). Hence, both **5** and **27** prevent the synergism of viral and pneumococcal NAs in A549 cells.

## Discussion

Since bacterial co-infection in influenza patients indicates a strong detrimental interaction between pneumococci and IAVs, targeting these two pathogens in parallel appears to be a promising avenue for the development of highly effective therapeutics. Thereby, addressing the issue of secondary pneumonia could also enhance the value of influenza prevention and treatment. Structural similarities of the surface protein NA, present on both *S. pneumoniae* and IAVs, render this enzyme a key target in this effort.

In this study, the flavonoid-rich TCM herbal drug sāng bái pí (white mulberry root bark) has been investigated from different angles (NA inhibition, cytotoxicity, anti-AIV and anti-*S. pneumoniae* activities, inhibition of viral replication in the presence of bacterial NAs) with different assay systems (both target- and cell-based) as potential source for natural anti-influenza virus and antipneumococcal compounds.

Our studies started from multicomponent mixtures (MAE and MAF). Earlier interference problems observed with NA assays prompted us to establish and apply a panel of complementary target-based and cell-based assays (Supplementary Tables S1 and S2). This panel allowed us to identify and confirm the distinct inhibitory activity of a fraction enriched with prenylated constituents (MAF) against the NA of pneumococci (Table 1) and influenza A viruses (Table 2 and Supplementary Table S3).

Among the compounds isolated from sāng bái pí multicomponent mixtures, the prenylated flavonoid derivatives **4** and **5** (Fig. 1) inhibited viral and pneumococcal NAs, pneumococcal growth and biofilm formation in the low micromolar range (Tables 1 and 2). As a herbal remedy against upper respiratory tract infections sāng bái pí is traditionally applied as decoction based on formulations containing several further plant materials, thereby, often relying on synergistic effects of their components. This makes scientific investigations of the active principle challenging. Because of our focus on prenylated flavonoids, compounds **1** to **7** were isolated in a target-oriented approach from a methanol extract (MAE) and its bioactive fraction, MAF. However, we also identified **1** to **7** as constituents in the aqueous decoction of sāng bái pí (MAD), not ruling out their involvement in the bioactivity of the traditional application form.

Follow-up studies with 27 congeners of **5** (non-, mono-, and di-prenylated; Fig. 2) revealed a direct correlation between the degree of prenylation and bioactivity. In full agreement with previously published data^8^, oseltamivir does neither inhibit planktonic growth nor biofilm formation of *S. pneumoniae* (Tables 1 and 2), despite its inhibitory activity on viral NA. In contrast, we observed a correlation between inhibitory activity on bacterial NA, bacterial growth and biofilm formation for compound **5** (and some of its di-prenylated congeners). However, a causative relationship between the inhibition of NA, bacterial growth and/or biofilm formation could not be conclusively established.

Congeners of **5** showed consistent, potent activities in all different types of *in vitro* assays. Compound **27** inhibited both viral and pneumococcal NA; it reduced IAV replication in presence and absence of NanA, *S. pneumoniae* growth in suspension, and biofilm formation. Whether di-prenylated congeners effectively engage in the complex interactions between IAV and pneumococci *in vivo* will require further studies in superinfection models. The data accumulated so far indicate that the investigated di-prenylated flavonoids could serve as a promising starting point for the development of new therapeutics able to disrupt the lethal synergism between IAV and *S. pneumoniae.*

## Methods

### Plant material

White mulberry root bark (in China also known under the name sāng bái pí) is the dried root bark of *Morus alba* L. (Moraceae). The material used for this study was purchased from Plantasia, Oberndorf, Austria. In TCM, the root is collected in late autumn while the leaves are falling off and in early spring before germination. It is removed from the yellowish-brown coarse bark and cut longitudinally. The root bark is stripped off and dried in the sun. The material’s quality and identity was macroscopically (appearance, texture, odour, taste, etc.) and microscopically (transverse section) checked according to the monograph “sāng bái pí” of the Pharmacopoeia of the Peoples Republic of China^29^. Voucher specimens (JR-20090112-A1) are deposited in the Herbarium of the Department of Pharmacognosy, University of Vienna, Austria.

### Extraction, isolation, and identification of pure compounds

Dried ground root bark of *M. alba* (2 kg) was macerated with methanol (7 L at 22 °C for 7 days). For an exhaustive extraction the procedure was repeated three times. The dried extract (MAE, 168.6 g) was separated by liquid-liquid partition into a methanol-water phase (CH_3_OH-H_2_O 1:1) and a petroleum ether phase (PE). The dried CH_3_OH-H_2_O phase (111.4 g) was roughly fractionated by silica gel CC (Merck silica gel 60 PF254, 350 g; 12.5 × 20 cm) using a step gradient of CH_2_Cl_2_-CH_3_OH-acetone (CH_2_Cl_2_; CH_2_Cl_2_-CH_3_OH 95:5; 90:10; 75:25; 50:50; 34:67; 25:75; 10:90; CH_3_OH; CH_3_OH-acetone 1:1; acetone) to give six fractions (A1-6). Fraction A4 (MAF, 15.32 g) was separated by High Speed Counter Current Chromatography (HSCCC, Pharma Tech Research PTR) (flow rate 1.0 mL/min) using CHCl_3_ as stationary phase and CH_3_OH-H_2_O as the mobile phase (CHCl_3_-CH_3_OH-H_2_O; 4:4:3) obtaining twenty fractions B1-20. Fraction B8 yielded the purified compound **3** (140.86 mg), and fraction B16 the pure compound **2** (160.25 mg). The combined fraction of B11-13 (130.12 mg) enriched with **4** and **6** was fractionated by liquid CC (Merck Lobar; pre-packed column size A (240-10); LiChroprep RP-18, 40–63 μm; H_2_O/acetonitrile from 70:30 to 60:40 within 24.0 min to 30:70 in another 64 min; from 30:70 to 2:98 within 4.0 min; isocratic 2:98 for 20.0 min) to afford pure compounds **4** (37.34 mg) and **6** (48.52 mg).

A3 (2.05 g) was fractionated via a Sephadex LH-20 (Pharmacia Biotech, Sweden; 1.5 × 100 cm) column with CH_3_OH as mobile phase to gain eighteen fractions D1-18. D14 (62.48 mg) was further separated by Lobar (column size A (240-10); LiChroprep RP-18, 40–63 μm) using H_2_O/acetonitrile as mobile phase (v/v; isocratic 65:35 for 5.0 min; from 65:35 to 40:60 within 60.0 min to 2:98 in another 60.0 min) to give 14.28 mg of **7** (14.28 mg). D12 (210.09 mg) and D13 (91.70 mg) were also separated by Lobar (column size A (240-10); LiChroprep RP-18, 40–63 μm; water/acetonitrile isocratic 40:60 for 12.0 min; from 40:60 to 25:75 within 60.0 min; isocratic 25:75 for 60.0 min; from 25:75 to 2:98 within 60.0 min) to yield the pure compound **1** (22.85 mg) and **5** (38.52 mg).

All isolated compounds (**1** to **7**) were identified by 1D and 2D NMR experiments (Bruker TXI600 NMR spectrometer operating at 600 MHz). The spectroscopic data of compounds **1** to **7** agreed with those published previously for sanggenon B (**1**), sanggenon C (**2**), sanggenon D (**3**), sanggenon G (**4**), sanggenol A (**5**), kuwanon L (**6**), and the 1:1 mixture of moracin O and moracin P (**7**)^30^,^31^,^32^,^33^,^34^,^35^,^36^,^37^. Their purity was checked using TLC and LC-MS (Bruker-Daltonics Esquire 3000 plus ion trap) and determined as >95% in all cases.

### Preparation of sāng bái pí water decoction (MAD) and HPLC comparison of MAD, MAE and MAF

Dried ground root bark of *M. alba* (10 g) was soaked exhaustively with cold water (0.5 L) for 1 h. Subsequently the material was decocted for 20 min in a pot with a lid. After filtration through cotton wool, the decoction procedure was repeated with the remaining plant material. The filtrated decoctions were combined (MAD) and an aliquot was used for HPLC analyses. HPLC of MAE, MAF, and MAD (Supplementary Fig. 1) was performed on a Shimadzu device consisting of an LC-10ADVP solvent delivery system, a FCV-10ALVP low-pressure gradient flow control valve, an SCL-10AVP system controller, a DGU-14A degasser, and a SPD-M20A photodiode array (PDA) detector. LC-parameters: stationary phase: Agilent Zorbax SB-C18 3.5 μm (150 × 4.6 mm); temperature: 25 °C; mobile phase: A = water; B = acetonitrile; flow rate 1.0 mL/min; PDA detection wavelength: 205 nm; injection volume: 10 μL; Separations were performed by gradient elution (70/30 A/B in 5 min to 55/45 A/B, then within 15 min to 45/55 A/B, and within 2 min to 2/98 A/B), followed by a 10 min column wash (2A/98B) and a re-equilibration period of 10 min. All chemicals and solvents used were analytical grade.

### Sanggenol A (5) congeners

Congeners of **5** were purchased from AnalytiCon Discovery GmbH, Potsdam, Germany. The purity of the compounds was ≥95%, except for **20**, **12**, and **26** where it was between 70–85%, as stated and certified by the vendor. Purity was determined by HPLC-ELSD or NMR.

### Cells, viruses, and bacteria

Madin-Darby canine kidney (MDCK) cells (Cat.nr. RIE 328, Friedrich-Loeffler Institute, Riems, Germany) were grown in Eagle’s minimum essential medium (EMEM), supplemented with 10% fetal bovine serum, 1% non-essential amino acids, 1 mM sodium pyruvate and 2 mM L-glutamine (Lonza, Basel, Switzerland). Serum-free EMEM containing 2 mM L-glutamine, 2 μg/mL trypsin and 0.1% sodium bicarbonate was used in antiviral assays. Human lung carcinoma cells (A549; Institute of Molecular Virology, University of Münster, Germany) were maintained in Dulbecco’s Modified Eagle Medium (Lonza Group Ltd, Basel, Switzerland), supplemented with 10% fetal calve serum (PAA Laboratories GmbH, Cölbe, Germany). Cells were cultivated at 37 °C with 5% CO_2_.

H1N1 influenza virus WSN/33 (amantadin-sensitive^25^) and A/Jena/8178/2009 (8178/09) were applied in the fluorescence (FL)-based neuraminidase (NA) inhibition (FL assay) as well as in the cytopathic effect (CPE) inhibition assay. Strain 8178/09 was additionally used to study the inhibitory effect of neuraminidase inhibitors (NAIs) in the presence and absence of bacterial neuraminidase (NA) in A549 cells. The hemagglutination-based NA inhibition assay (HA assay) was performed with Jena/5258^38^.

Reference strain DSM20566 (serotype 1; ATCC 33400, Leibniz Institute DSMZ-German Collection of Microorganisms and Cell Cultures, Heidelberg, Germany) was grown on Columbia blood agar plates supplemented with 5% sheep blood (Becton Dickinson GmbH, Heidelberg, Germany) at 37 °C in an atmosphere enriched with 5% CO_2_ overnight. During pre-cultivation, bacteria were grown in brain heart infusion (BHI) broth^8^.

### Enzyme inhibition assays

The FL assay was performed as described recently^13^ using the substrate 20-(4-methylumbelliferyl)-a-D-N-acetylneuraminic acid (MUNANA). The inhibition of the NA of whole 8178/09 and WSN/33 preparations and recombinant NanA of pneumococcus DSM20566^3^ were studied. The readout was performed with a microplate reader (FLUOstar Omega, BMG Labtech GmbH, Ortenberg, Germany). The highest tested compound concentration was 100 μM. The 50% inhibitory concentration (IC_50_) values were calculated with the JASPR curve fitting software^39^. A minimum of three independent experiments was conducted to calculate means and standard deviations.

The inhibitory effects of the studied compounds on viral NA (Jena/5258) and pneumococcal NA (precipitated bacterial proteins of DSM20566) were also evaluated with HA assays, which were described recently^13^. In the viral HA assay, hemagglutinin of strain 5258/09 agglutinates human erythrocytes at 4 °C. The elution of agglutinated erythrocytes by the viral NA activity at 37 °C can be blocked by inhibitors^13^. Strain 5258/09 had to be used because neither WSN/33 nor 8178/09 were able to elute agglutinated erythrocytes. In the bacterial HA assay, the NA of DSM20566 cleaved sialic acid at the cell membrane, thereby providing binding sites for peanut lectin. Binding of lectin leads to hemagglutination of erythrocytes. Inhibition of pneumococcal NAs circumvents hemagglutination^13^.

Oseltamivir carboxylate GS4071 (oseltamivir; Roche AG, Basel, Switzerland) was included as reference compound in both assays. It was dissolved in water as 10 mM stock solutions.

### Determination of cytotoxicity and antiviral activity

The 50% cytotoxic concentration (CC_50_) was determined on two-day-old confluent MDCK and three-day-old A549 cell monolayers grown on 96-well plates 72 h after adding the test compounds (maximum tested concentration: 100 μM; threefold dilutions) as described previously^8^,^40^. Cytopathic effect (CPE) inhibition was measured 48 h after 8178/09 and WSN/33 infection when a multiplicity of infection of 0.01/TCID_50_ (50% tissue culture infection dose) per cell resulted in a complete CPE of untreated MDCK cells. Same concentrations as in cytotoxicity assay were studied. The testing conditions were published in detail earlier^40^. The experimental conditions for antiviral experiments without and with pneumococcal NanA in A549 cells were described recently^3^.

The mean CC_50_ and IC_50_ values and standard derivations were calculated from at least 3 individual experiments.

### Inhibition of bacterial planktonic growth and biofilm formation

Broth microdilution assay and biofilm assay were conducted as described previously^8^. Briefly, pneumococci of McFarland 0.5 (1.5 × 10^8^ colony forming unit (cfu)/mL) were 50× diluted in brain heart infusion broth (BHI), for microdilution assay. Compounds were mixed with bacteria in a 96-well V-shape plate and incubated overnight at 37 °C with 5% CO_2_. The highest compound concentration tested was 50 μM (twofold dilutions). The growth of bacteria was monitored by measuring optical density at 620 nm. The minimal inhibitory concentration (MIC_90_) was defined as the drug concentration that reduced 90% turbidity of the untreated bacterial suspension.

For the biofilm assay, broth was diluted 50-fold in tryptic soy broth and incubated for 2 h at 37 °C with 5% CO_2_ in 96-well F-bottom plates. Then, the supernatant was replaced by 200 μL of fresh medium without or with compound (maximum 50 μM; each concentration in duplicate). After 24 h incubation, plates were washed with water and stained with crystal violet overnight. After rinsing the plates with water, the crystal violet was eluted with lysis buffer^40^, and the optical density of the elution was measured at 550 nm. Minimal biofilm inhibitory concentration (MBIC_90_) was defined as the drug concentration reducing the optical density of untreated controls by 90%.

### Scanning electron microscopy studies

Overnight plate cultures of DSM20566 were inoculated in tryptic soy broth (TSB) and grown to mid-logarithmic phase at 37 °C in 5% CO_2_. Bacteria were diluted in TSB to McFarland 0.5 (~1.5 × 10^8^ cfu/mL) and inoculated onto glass slides (diameter of 12 mm) placed on the floor of wells of a 24-well plate. Three hours after inoculation supernatant was replaced by fresh medium without or with **27** or oseltamivir (both 10 µM). Then bacteria were incubated at 37 °C and 5% CO_2_ for 7 d. After fixation with 2.5% glutaraldehyde in cacodylate buffer (0.1 M, pH 7.2) for 24 h, the glass slides with bacteria were washed three times with cacodylate buffer and dehydrated with ascending ethanol concentrations (10, 30, 50, 70, 90, and 100%). Subsequently, the samples were critical-point dried using liquid CO_2_ (CPD 300, Leica, Wetzlar, Germany) and gold sputtered (SCD 005, BAL-TEC, Liechtenstein) to prevent surface charging. Finally, the samples were examined with a field emission gun scanning electron microscope LEO-1530 (Zeiss, Oberkochen, Germany). For studying the time-dependent inhibitory effect on biofilm formation, **27** was added to the medium at 3, 6, 9 or 48 h after DSM20566 inoculation.

### Statistical analyses

Mean and standard deviation (SD) values were analysed using Microsoft Excel 2010. In the studies on the interference of small molecules with the FL assay the cut-off value was set at mean ± 2 SD of control (without compound). Statistically significant differences were analysed by using Welch’s t-test (Microsoft Excel).

## Additional Information

**How to cite this article**: Grienke, U. *et al*. Discovery of prenylated flavonoids with dual activity against influenza virus and Streptococcus pneumoniae. *Sci. Rep.* **6**, 27156; doi: 10.1038/srep27156 (2016).

## Supplementary Material

Supplementary Information

Supplementary Information

## Figures and Tables

**Table 1:**
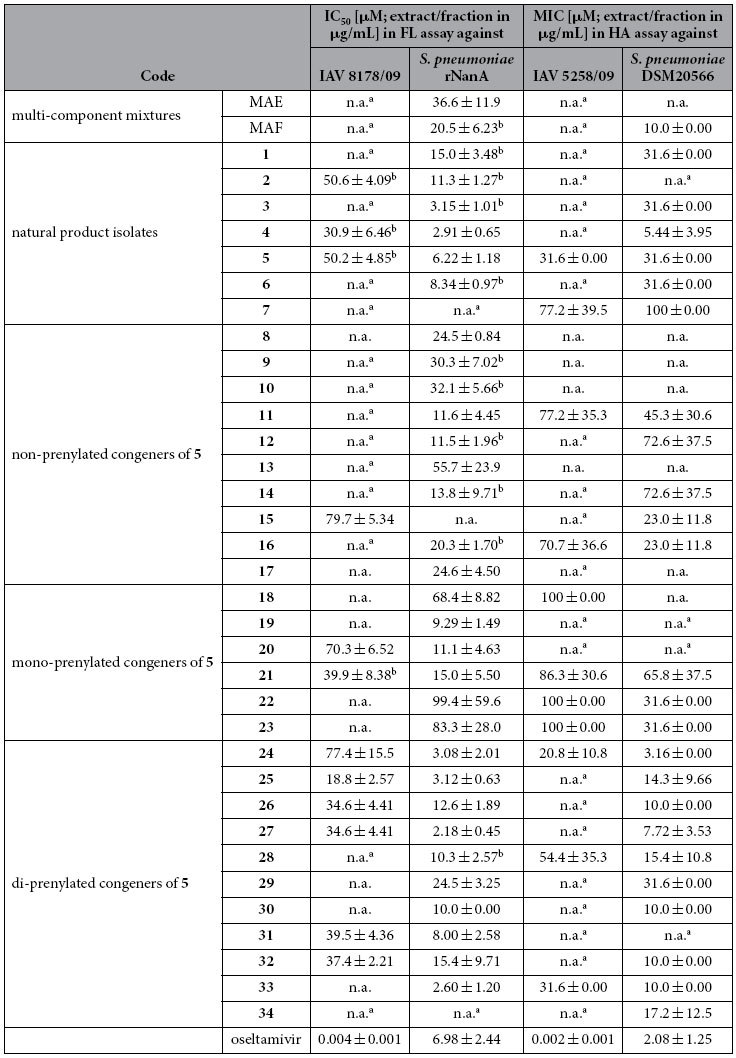
Inhibition of influenza A virus (IAV) and *Streptococcus pneumoniae* neuraminidases (NAs) by the *M. alba* root bark methanol extract (MAE), its fraction enriched with prenylated constituents (MAF), *M. alba* isolates and congeners of sanggenol A (**5**).

The 50% inhibitory concentration (IC_50_) was determined with the IAV Jena/8178/2009 (8178/09) and recombinant NanA of the *S. pneumoniae* strain DSM20566 (rNanA) and the minimal inhibitory concentration (MIC) with IAV Jena/5258/09 (5258/09) and total protein of *S. pneumoniae* DSM20566 in fluorescence (FL)-based and hemagglutination (HA)-based NA inhibition assays, respectively.

^a^No activity (n.a.) was found at concentrations where compounds were without self-fluorescence (Supplementary Table S1), lyse, or hemagglutinate human erythrocytes (Supplementary Table S2).

^b^Self-fluorescence was observed (Supplementary Table S1). The highest tested concentration was 100 μg/mL (extract/fraction) or 100 μM (pure compounds). Means and standard deviations were determined in at least three independent experiments.

**Table 2:**
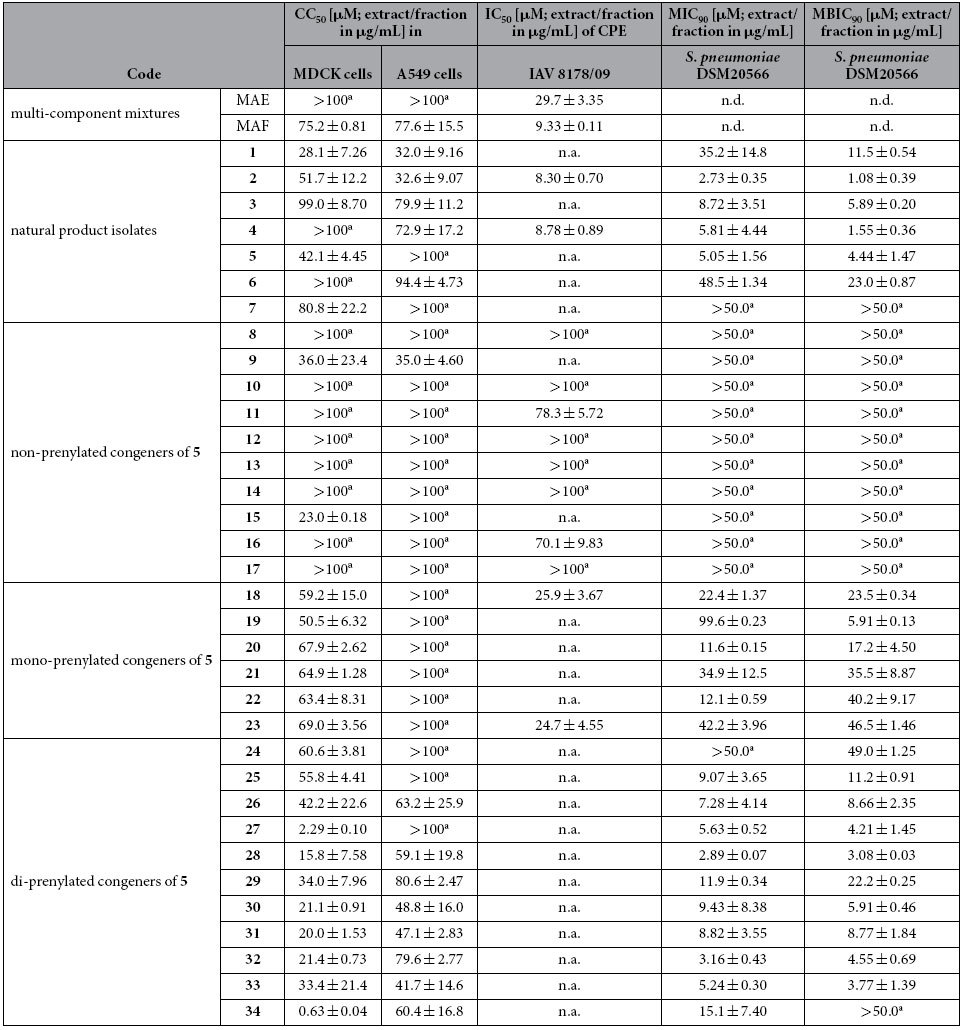
Cytotoxicity, anti-influenza A virus (IAV), and anti-*Streptococcus pneumoniae* activity of *M. alba* root bark methanol extract (MAE), its fraction enriched with prenylated constituents (MAF), *M. alba* isolates and congeners of sanggenol A (**5**).

Their 50% cytotoxic concentration (CC_50_) in Madin-Darby canine kidney (MDCK) cells and in human lung carcinoma cells (A549), their 50% inhibition concentration (IC_50_) determined against IAV Jena/8178/2009 (8178/09) in cytopathic effect (CPE) inhibition assay in MDCK cells as well as their effect on pneumococcal (strain DSM20566) growth (MIC_90_ = 90% minimal inhibitory concentration) and biofilm formation (MBIC_90_ = 90% minimal biofilm inhibitory concentration) is presented.

^a^highest concentration tested. Means and standard deviation of the extracts and compounds based on minimum of three independent assays. n.a., not active at noncytotoxic concentrations in MDCK cells (used in CPE inhibitory assay). n.d., not determined.
