# Supplementary figures and images for: Discovery of prenylated flavonoids with dual activity against influenza virus and Streptococcus pneumoniae
Source: Sci Rep. 2016 Jun 3;6:27156. doi: 10.1038/srep27156 (PMC4891693; doi:10.1038/srep27156)

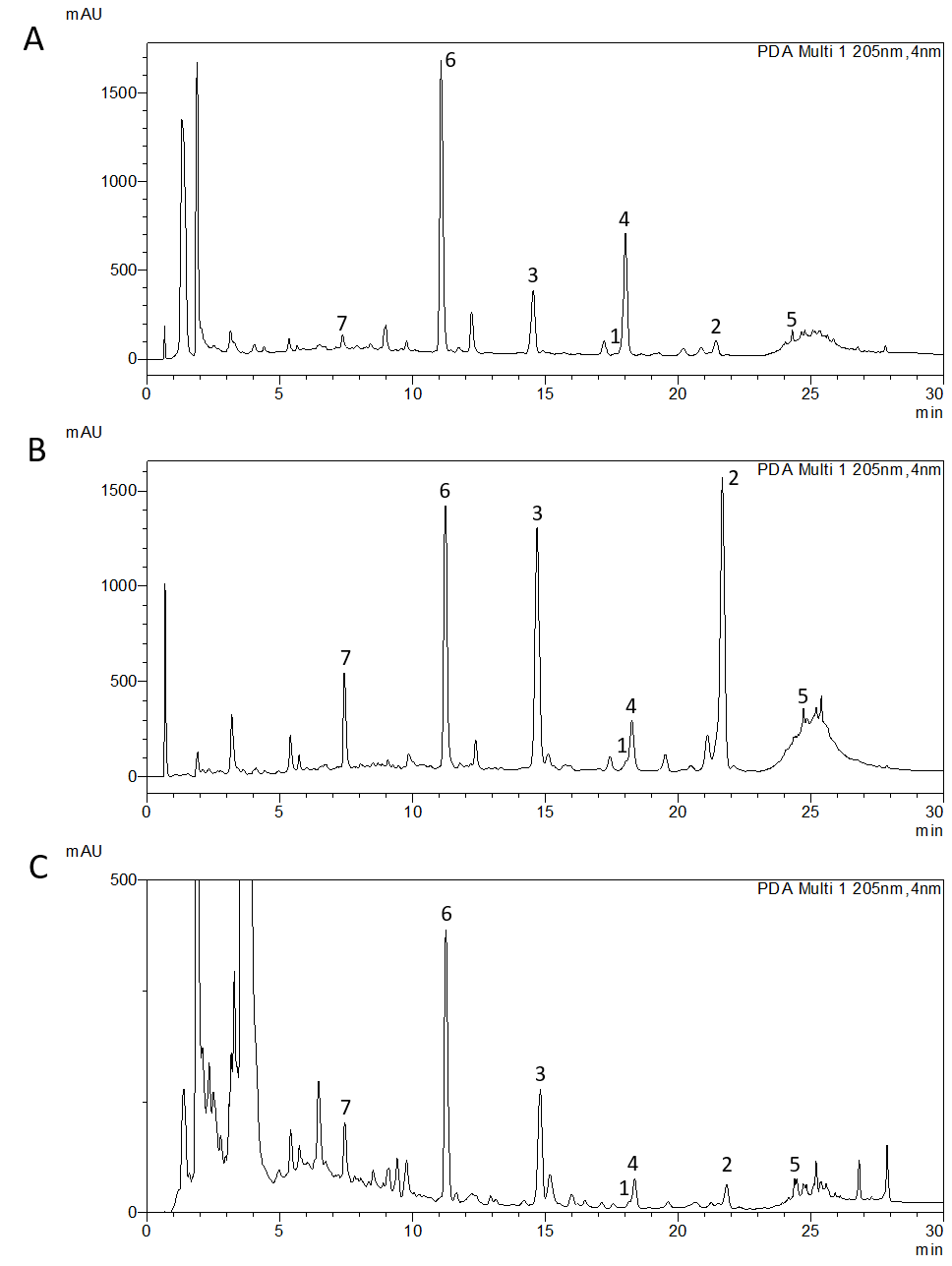

Supplement: Supplementary Information [file srep27156-s1.tiff]
