# Supplementary material for: Discovery of prenylated flavonoids with dual activity against influenza virus and Streptococcus pneumoniae
Source: Sci Rep. 2016 Jun 3;6:27156. doi: 10.1038/srep27156 (PMC4891693; doi:10.1038/srep27156)
Supplement: Supplementary Information [file srep27156-s2.pdf]

Supporting Information to

Discovery of prenylated flavonoids with dual activity against influenza virus and Streptococcus pneumoniae

Ulrike Grienke[1+], Martina Richter[2+], Elisabeth Walther[2], Anja Hoffmann[2], Johannes Kirchmair[3], Vadim Makarov[4], Sandor Nietzsche[5], Michaela Schmidtke[2*], Judith M. Rollinger[1*]

[1] Department of Pharmacognosy, Faculty of Life Sciences, University of Vienna, Althanstraße 14, 1090 Vienna, Austria

[2] Jena University Hospital, Department of Virology and Antiviral Therapy, Hans-Knoell-Strasse 2, 07745 Jena, Germany

[3] University of Hamburg, Center for Bioinformatics, Bundesstrasse 43, 20146 Hamburg, Germany

[4] A.N. Bakh Institute of Biochemistry RAS, Leninsky prospekt, 33, build. 2, Moscow, 119071, Russia

[5] Jena University Hospital, Center of Electron Microscopy, Ziegelmuehlenweg 1, 07743 Jena, Germany

Table S1. Self-fluorescence or quenching of test compounds.

	code	sample concentrations [µM; extract/fraction in µg/mL] with self-fluorescence[a]	quenching
multi-component mixtures	MAE	100	no[a]
	MAF	> 1	no[a]
natural product isolates	**1**	> 10	no[a]
	**2**	> 10	no[a]
	**3**	> 1	no[a]
	**4**	> 10	no[a]
	**5**	> 10	no[a]
	**6**	> 1	no[a]
	**7**	> 1	no[a]
non-prenylated congeners of **5**	**8**	no	no[b]
	**9**	> 1	no[b]
	**10**	> 1	no[b]
	**11**	100	no[b]
	**12**	> 1	no[b]
	**13**	100	no[b]
	**14**	> 1	no[b]
	**15**	no	no[b]
	**16**	> 1	no[b]
	**17**	no	no[b]
mono-prenylated congeners of **5**	**18**	no	no[b]
	**19**	no	no[b]
	**20**	no	no[b]
	**21**	100	no[b]
	**22**	no	no[b]
	**23**	no	no[b]
di-prenylated congeners of **5**	**24**	no	no[b]
	**25**	no	no[b]
	**26**	no	no[b]
	**27**	no	no[b]
	**28**	> 1	no[b]
	**29**	no	no[b]
	**30**	no	no[b]
	**31**	no	no[b]
	**32**	no	no[b]
	**33**	no	no[b]
	**34**	> 10	no[b]

[a] highest tested concentration 100 µM or 100 µg/mL in case of extract or fraction.
[b] highest tested concentration 10 µM.

Table S2. Interference of test compounds with hemagglutination (HA) of human erythrocytes.

code		sample concentrations [µM; extract/fraction in µg/mL] provoking in viral HA assay			bacterial HA assay	
		**induction of hem-agglutination[a]**	**prevention of virus-induced hemagglutination[b]**	**induction of erythrocyte lysis[c]**	**induction of hem-agglutination[d]**	**induction of erythrocyte lysis[e]**
multi-component mixtures	MAE	10-100	no	no	no	no
	MAF	3.16-100	no	no	no	no
natural product isolates	**1**	100 or no	31.6	100 or no	no	100
	**2**	no	3.1-100	10-100	no	31.6-100
	**3**	no	no	31.6-100	no	100
	**4**	no	3.1-100	10-100	no	31.6-100
	**5**	no	100 or no	100 or no	no	100
	**6**	no	31.6	100	no	100
	**7**	no	no	no	no	no
non-prenylated congeners of 5	**8**	no	no	no	no	no
	**9**	no	no	no	no	no
	**10**	no	no	no	no	no
	**11**	no	no	no	no	no
	**12**	10-100	no	no	no	no
	**13**	no	no	no	no	no
	**14**	10-100	no	no	no	no
	**15**	3.16-100	no	no	no	no
	**16**	no	no	no	100	no
	**17**	100	no	no	no	no
mono-prenylated congeners of 5	**18**	no	no	no	no	no
	**19**	100	no	no	no	31.6-100
	**20**	no	no	100 or no	no	100 or no
	**21**	no	no	no	no	no
	**22**	no	no	no	no	no
	**23**	no	no	no	no	no
di-prenylated congeners of 5	**24**	no	100	no	no	100
	**25**	no	31.6	100 or no	no	100
	**26**	31.6-100	no	31.6-100	no	100
	**27**	10-100	3.16-100	no	no	31.6-100
	**28**	no	no	no	no	100
	**29**	no	31.6-100	100	no	100
	**30**	100 or no	10-100	no	no	31.6-100
	**31**	no	no	31.6-100	no	100
	**32**	no	no	31.6-100	no	31.6-100
	**33**	100	no	no	no	no
	**34**	10-100	no	no	no	no

[a] Test compound induced a hemagglutination of human erythrocytes (2 h at 4 °C). [b] Test compound prevented the virus-induced hemagglutination of human erythrocytes (2 h at 4 °C). [c] Test compounds lysed the human erythrocytes (2 h at 4 °C). [d] Test compound induced a hemagglutination of human erythrocytes in the presence of lectin (24 h, 4 °C). [e] Test compounds lysed the human erythrocytes (4 h at 37 °C and 12 h at 4 °C).

Table S3. Inhibition of influenza virus A/WSN/33 NA in fluorescence-based NA inhibition assay (FL assay) and cytopathic effect (CPE assay) in MDCK cells by the *M. alba* root bark MeOH extract (MAE), its fraction enriched with prenylated constituents (MAF), *M. alba* isolates, and congeners of sanggenol A (5).

code		IC_{50} [µM; extract/fraction in µg/mL] in FL assay	IC_{50} [µM; extract/fraction in µg/mL] of CPE assay
multi-component mixtures	MAE	62.0 ± 8.03[a]	76.6 ± 2.58
	MAF	>1[a]	23.0 ± 3.44
natural product isolates	**1**	31.2 ± 10.1[a]	n.a.
	**2**	9.18 ± 1.50[a]	n.a.
	**3**	31.2 ± 10.1[a]	n.a.
	**4**	61.7 ± 17.8[a]	15.2 ± 14.0
	**5**	65.5 ± 9.29[a]	35.4 ± 14.6
	**6**	37.2 ± 10.6[a]	69.8 ± 6.81
	**7**	>1[a]	n.a.
non-prenylated congeners of 5	**8**	>100	>100
	**9**	>100[a]	n.a.
	**10**	>100[a]	>100
	**11**	>100[a]	68.4 ± 12.9
	**12**	77.7 ± 5.56	81.3 ± 10.4
	**13**	76.8 ± 9.64	>100
	**14**	>100[a]	>100
	**15**	>100	n.a.
	**16**	62.0 ± 8.03[a]	>100
	**17**	9.18 ± 1.50[a]	>100
mono-prenylated congeners of 5	**18**	>100	11.4 ± 3.00
	**19**	>100	n.a.
	**20**	>100	n.a.
	**21**	45.54 ± 6.06	n.a.
	**22**	>100	n.a.
	**23**	27.0 ± 3.24	n.a.
di-prenylated congeners of 5	**24**	>100	n.a.
	**25**	41.4 ± 6.98	n.a.
	**26**	63.9 ± 11.1	n.a.
	**27**	33.1 ± 11.7	n.a.
	**28**	>100[a]	n.a.
	**29**	53.1 ± 17.5	n.a.
	**30**	62.5 ± 5.40	n.a.
	**31**	37.9 ± 14.6	n.a.
	**32**	26.9 ± 1.85	n.a.
	**33**	97.7 ± 0.46	n.a.
	**34**	>100[a]	n.a.
control	oseltamivir	0.0016 ± 0.0001	n.d.

[a] Compounds exerted self-FL at certain concentrations (see Table S1). n.a. not active at noncytoxic concentrations in MDCK cells (Table 2). The highest tested concentration was 100 µg/mL or 100 µM for extracts and compounds, respectively. n.d. not determined

Table S4. Product numbers, Chemical Abstracts Service (CAS) registry numbers, and names of investigated congeners of sanggenol A (5).

code	product number (AnalytiCon Discovery)	CAS registry number	name
**8**	NP-001206	211178-43-7	2,3-dihydro-5,7-dihydroxy-6-methoxy-2-phenyl-4H-1-benzopyran-4-one
**9**	NP-001581	69097-98-9	(±)-homoeriodictyol
**10**	NP-003773	69097-99-0	(±)-hesperetin
**11**	NP-001578	4049-38-1	(±)-eriodictyol
**12**	NP-012970	121694-88-0	2',4',5,7-tetrahydroxyflavanone
**13**	NP-013702	80366-15-0	3,5,7,2',6'-pentahydroxyflavanone
**14**	NP-012592	144707-17-5	2',3',4',5,7-pentahydroxyflavone
**15**	NP-000380	491-70-3	luteolin
**16**	NP-010620	480-16-0	morin
**17**	NP-004431	36804-17-8	(±)-silybin
**18**	NP-013030	886620-61-7	(±)-abyssinone II
**19**	NP-010957	913691-36-8	INDEX NAME NOT YET ASSIGNED
**20**	NP-002011	93766-65-5	4',5,7-trihydroxy-3'-prenylflavanone
**21**	NP-012960	183997-08-2	2,3-dihydro-5,7-dihydroxy-2-[4-hydroxy-3-methoxy-5-(3-methyl-2-butenyl)phenyl]-4H-1-benzopyran-4-one
**22**	NP-003517	1083197-70-9	2,3-dihydro-5,7-dihydroxy-2-(4-hydroxy-3-methoxyphenyl)-6-(3-methyl-2-buten-1-yl)-4H-1-benzopyran-4-one
**23**	NP-005230	1246094-70-1	(2R,3R)-rel-4H-2,3-dihydro-3,5,7-trihydroxy-2-(4-hydroxyphenyl)-8-(3-methyl-2-buten-1-yl)-1-benzopyran-4-one
**24**	NP-015140	67832-07-9	(R,S)-8-geranyl-5,7-dihydroxyflavanone
**25**	NP-009005	910619-01-1	INDEX NAME NOT YET ASSIGNED
**26**	NP-009004	96917-35-0	bonanniol A
**27**	NP-012357	201480-12-8	abyssinone-V 4'-methyl ether
**28**	NP-000563	68236-11-3	6,8-diprenylnaringenin
**29**	NP-006454	952647-85-7	2-(3,5-dihydroxyphenyl)-2,3-dihydro-5,7-dihydroxy-6,8-bis(3-methyl-2-buten-1-yl)-4H-1-benzopyran-4-one
**30**	NP-001083	51225-28-6	6,8-diprenylgenistein
**31**	NP-013070	66777-70-6	6,8-diprenylorobol
**32**	NP-012233	211183-29-8	orientanol E
**33**	NP-016087	1246094-68-7	2,4-bis(3-methyl-2-buten-1-yl)-5-(2-phenylethyl)-1,3-benzenediol
**34**	NP-018637	28233-35-4	atalphylline

Figure S1. HPLC analysis of the *M. alba* root bark MeOH extract (MAE) (A), its fraction enriched with prenylated constituents (MAF) (B), and a decoction (MAD) (C) produced according to the traditional Chinese medicine.

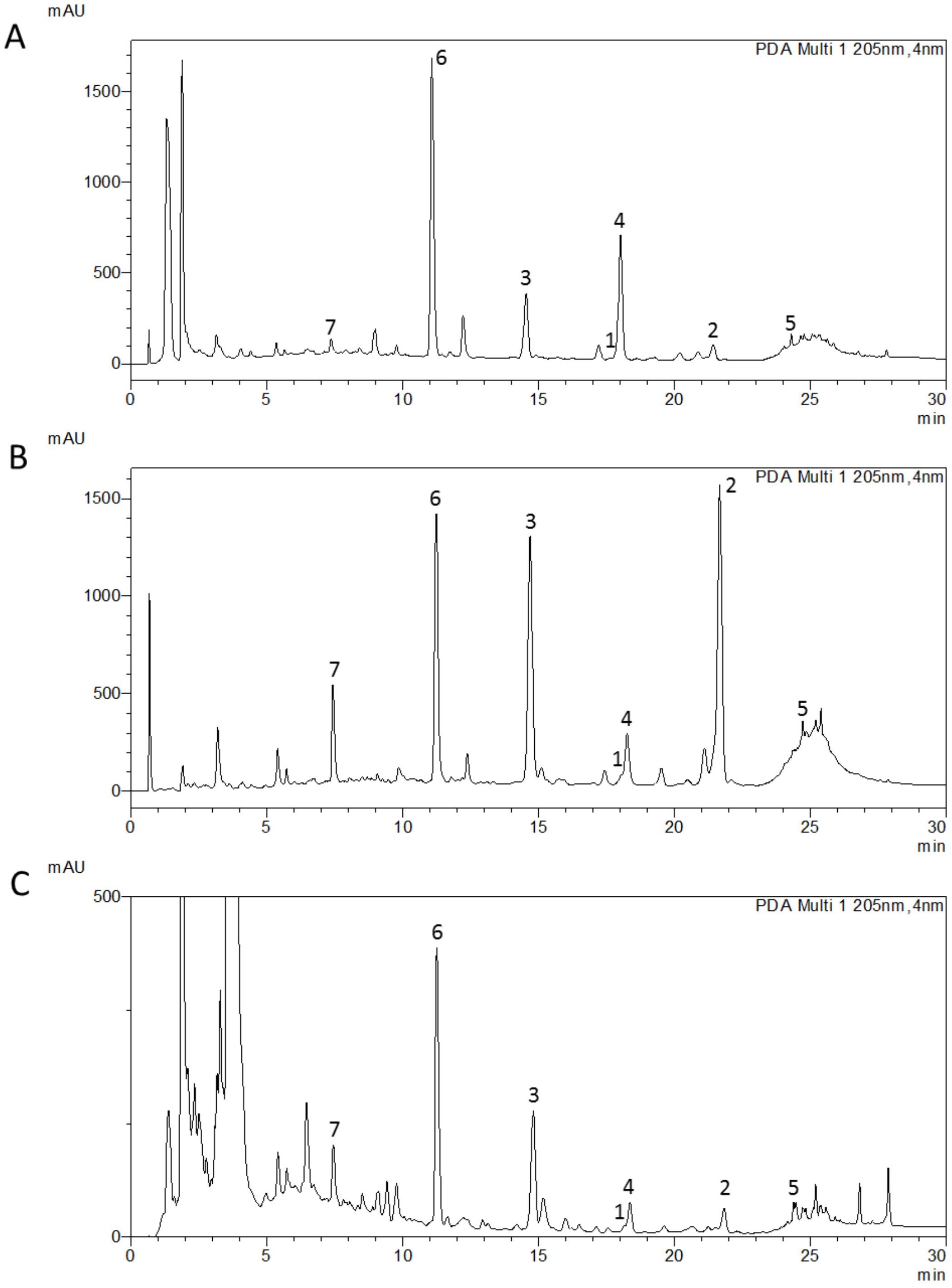

HPLC was performed on Shimadzu device consisting of an LC-10ADVP solvent delivery system, a FCV-10ALVP low-pressure gradient flow control valve, an SCL-10AVP system controller, a DGU-14A degasser, and a SPD-M20A photodiode array (PDA) detector. LC-parameters: stationary phase: Agilent Zorbax SB-C18 3.5µm (150 x 4.6 mm); temperature: 25 °C; mobile phase: A = water; B = acetonitrile; flow rate 1.0 mL/min; PDA detection wavelength: 205 nm; injection volume: 10 µL; Separations were performed by gradient elution (70/30 A/B in 5 min to 55/45 A/B, then within 15 min to 45/55 A/B, and within 2 min to 2/98 A/B), followed by a 10 min column wash (2A/98B) and a re-equilibration period of 10 min. All chemicals and solvents used were analytical grade.
